# Do self- reported intentions predict clinicians' behaviour: a systematic review

**DOI:** 10.1186/1748-5908-1-28

**Published:** 2006-11-21

**Authors:** Martin P Eccles, Susan Hrisos, Jill Francis, Eileen F Kaner, Heather O Dickinson, Fiona Beyer, Marie Johnston

**Affiliations:** 1 Centre for Health Services Research, University of Newcastle upon Tyne, 21 Claremont Place, Newcastle upon Tyne, NE2 4AA, UK; 2 Health Services Research Unit, University of Aberdeen, Health Sciences Building, Foresterhill, Aberdeen AB25 2ZD, UK; 3 Department of Psychology, University of Aberdeen, Health Sciences Building, Foresterhill, Aberdeen AB25 2ZD, UK

## Abstract

**Background:**

Implementation research is the scientific study of methods to promote the systematic uptake of clinical research findings into routine clinical practice. Several interventions have been shown to be effective in changing health care professionals' behaviour, but heterogeneity within interventions, targeted behaviours, and study settings make generalisation difficult. Therefore, it is necessary to identify the 'active ingredients' in professional behaviour change strategies. Theories of human behaviour that feature an individual's "intention" to do something as the most immediate predictor of their behaviour have proved to be useful in non-clinical populations. As clinical practice is a form of human behaviour such theories may offer a basis for developing a scientific rationale for the choice of intervention to use in the implementation of new practice. The aim of this review was to explore the relationship between intention and behaviour in clinicians and how this compares to the intention-behaviour relationship in studies of non-clinicians.

**Methods:**

We searched: PsycINFO, MEDLINE, EMBASE, CINAHL, Cochrane Central Register of Controlled Trials, Science/Social science citation index, Current contents (social & behavioural med/clinical med), ISI conference proceedings, and Index to Theses. The reference lists of all included papers were checked manually. Studies were eligible for inclusion if they had: examined a clinical behaviour within a clinical context, included measures of both intention and behaviour, measured behaviour after intention, and explored this relationship quantitatively. All titles and abstracts retrieved by electronic searching were screened independently by two reviewers, with disagreements resolved by discussion.

**Discussion:**

Ten studies were found that examined the relationship between intention and clinical behaviours in 1623 health professionals. The proportion of variance in behaviour explained by intention was of a similar magnitude to that found in the literature relating to non-health professionals. This was more consistently the case for studies in which intention-behaviour correspondence was good and behaviour was self-reported. Though firm conclusions are limited by a smaller literature, our findings are consistent with that of the non-health professional literature. This review, viewed in the context of the larger populations of studies, provides encouragement for the contention that there is a predictable relationship between the intentions of a health professional and their subsequent behaviour. However, there remain significant methodological challenges.

## Background

Implementation research is the scientific study of methods to promote the systematic uptake of clinical research findings into routine clinical practice, and hence to reduce inappropriate care. It includes the study of influences on healthcare professionals' behaviour, and methods to enable them to use research findings more effectively. Over the last decade a considerable body of such research has been both published and reviewed [1,2]. This research demonstrates that several (variously complex) interventions can be effective in changing health care professionals' behaviour. However, these studies have substantial heterogeneity within interventions, targeted behaviours, and study settings that render generalisability problematic [3].

In order to optimise the number of costly and time consuming future trials that need to be conducted – and to enhance their generalisability, it is necessary to identify the 'active ingredients' in professional behaviour change strategies. Interventions could be effective at changing behaviour for two reasons: they may contain components that are always effective in changing any behaviour, or they may contain components that overcome specific barriers encountered in relation to a particular behaviour. Hence, two approaches are necessary to identify the 'active ingredients' in the complex interventions of implementation trials: 1) Develop an understanding of the factors underlying professional behaviour in order to identify what sorts of processes should be targeted by interventions (process modelling) [4], and 2) Develop an understanding of how the interventions work and can be optimised (intervention modelling) [5].

In intervention modelling, key elements of an intervention are manipulated in a manner that simulates a real situation as much as possible, and interim endpoints are measured rather than changes in professional behaviour or healthcare outcome. A typical interim endpoint is a stated intention to behave in a particular way. Intention has been defined as "indications of how hard people are willing to try, of how much effort they are planning to exert, in order to perform a behaviour" [6] (see page 181). Compared to large scale trials, modelling experiments have two potential advantages – smaller size and shorter timescales – whilst still offering experimental control. Although proof of concept work has demonstrated the feasibility of such studies [5], a prerequisite of this method is a predictable link between the interim endpoints and changes in actual professional behaviour. For the method to be valid, interim endpoints (e.g. measures of intention) must be predictive of real world outcomes. This is the case for behavioural intention in non-clinical populations as demonstrated by reviews of both observational and experimental studies. Godin and Kok [7] reported averaged correlations between intention and different health-related behaviours ranging from 0.35 to 0.56 (i.e. intention was accounting for between 12% and 31% of the variance in behaviour). Armitage and Connor [8], using 63 independent studies reporting prospectively measured behavioural data, reported that the Theory of Planned Behaviour (TPB) variables that directly influence behaviour (intention and perceived behavioural control) accounted for a similar proportion of the variance in behaviour. When behavioural measures were self-reported, the TPB accounted for more of the variance in behaviour than when behaviour measures were objective or observed. A meta-analysis of 10 meta-analyses by Sheeran [9] reported that intention accounted for almost one-third of the variance in behaviour. Finally, Webb and Sheeran [10] reviewed experimental studies to relate change in intention to change in behaviour. From a meta-analysis of 47 experimental tests of the intention-behaviour relationship, they concluded that a "medium-to-large" change in intention leads to a "small-to-medium" change in behaviour. None of these reviews [7-10] specifically identified studies of healthcare professionals and clinical behaviours.

These reviews demonstrate that there is a reliable, but not a perfect, relationship between stated intention and behaviour. Considerable research efforts have been directed to addressing the 'intention-behaviour gap' and two approaches have been proposed. One addresses the variability of the link by focusing on moderators of the intention-behaviour relationship, such as intention certainty and attitudinal versus normative control [11]. According to this approach, it is possible to predict which individuals will enact their intentions (e.g. those whose intentions are attitudinally controlled). A second approach focuses on mediators of the intention-behaviour relationship, or processes that might be regarded as 'post-intentional,' such as implementation intentions [12], action plans and coping plans [13]. This approach identifies processes that assist individuals to enact their intentions, thereby minimising the size of the intention-behaviour gap.

It has been argued [14] that the intentions and behaviour of clinicians are influenced by measurable psychological variables (e.g. attitudes) in the same way as the intentions and behaviour of any individual. However, most psychological models of behaviour are predicated on the basis of perceived consequences of a behaviour being experienced by the actor (e.g. *If I give up smoking, I will improve my physical fitness*). For clinicians, the perceived (or actual) consequences of their clinical behaviours are often (though not always) experienced by another person (e.g. *If I lower my patient's blood pressure, she will have a reduced risk of premature mortality*). Furthermore, clinical behaviour is constrained by a number of factors, such as imperatives dictated by a professional role, legal responsibilities, and principles of clinical governance. Although in a theoretical context these may be seen as control factors that influence behaviour together with intentions, some of these factors may not be articulated by clinicians. As they always form part of the context of any clinical behaviour, they may be implicit but powerful influences on intentions and behaviour, or the relationship between them. Therefore, it is important to explore the intention-behaviour relationship relating to clinical behaviour.

This review addresses the following two questions: 1) What is the nature of the relationship between measures of intention and clinical behaviours in clinicians? and 2) How does this compare to the intention-behaviour relationship in studies of non-clinicians?

## Methods

### Criteria for studies included in the review

We included any study that examined clinical behaviour (behaviour enacted by a clinician (doctors, nurses, and allied health professionals) within a clinical context with respect to a patient or their care. We also included measures of both intention and behaviour and explored this relationship quantitatively, and measured behaviour after intention had been measured.

### Measures of intention and behaviour

As clinical behaviour is often enacted in contexts with a high focus on patient privacy, confidentiality, and matters of personal sensitivity, it may not always be feasible to measure clinical behaviour through direct independent observation. Where direct measurement is not possible, studies may use alternative or "reported" measures of the behaviour under investigation. The following criteria were used to define acceptable measures of intentions, observed behaviour, and reported behaviour for studies included in this review.

Two methods of measuring intention were included:

1. Strength of intention, where clinicians are asked to indicate (e.g., on a 7-point scale) how strongly they agree or disagree with a specific intention statement regarding the target behaviour. Intention could also be characterised as 'willingness' or 'readiness.'

2. Percentage estimates, where clinicians are asked to indicate for what proportion of patients with a particular condition they intend to perform the target behaviour.

Four measures of behaviour were included:

1. Self-reported behaviour, where, for example, a clinician completes a diary or questionnaire, recording how many patients with type 2 diabetes were seen that day or that week – and how many of the patient's feet were inspected. Self-report of behaviour must be reported retrospectively, i.e. after the behaviour has been enacted, and must be measured after intention has been measured.

2. Observed behaviour, including directly observed, audio or video-taped behaviour.

3. Patient-reports of clinician behaviour, where, for example, a patient is asked to report which prescribed medications are being taken as a measure of the clinician's prescribing behaviour.

4. Documented behaviour, where, for example, the ordering of a radiology test is automatically recorded as part of the ordering process, or a blood pressure reading is recorded in a patient's case notes by the clinician.

### Search strategy

The following databases were searched: PsycINFO (1840-Aug 2004), MEDLINE (1966-Aug wk 3 2004), EMBASE (1980-Aug wk 34), CINAHL (1982-Aug wk 3 2004), Cochrane Central Register of Controlled Trials (2004 issue 2), Science/Social science citation index (1970-Aug 2004), Current contents (social & behavioural med/clinical med) (1998-Aug 2004), ISI conference proceedings (1990-Aug 2004), and Index to Theses (1716-Aug 2004). The search terms for intention, behaviour, health professionals, and scenarios are shown in Table 1. The search domains were combined as follows: (Intention) AND (Behaviour) AND (Health professionals), (Intention-behaviour) AND (Health professionals), (Behaviour) AND (Outcomes) AND (Health professionals). The reference lists of all included papers were checked manually.

**Table 1:** Keyword combinations for four domains, combined for the database search

Intention (Intention or intend*)	Behaviour near behavio?r*	Health professionals
**Thesaurus heading:**	**Thesaurus headings:**	**Thesaurus headings:**
**INTENTION**	• **BEHAVIOR**	• **HEALTH PERSONNEL**
	• **CHOICE BEHAVIOR**	• **ATTITUDE OF HEALTH PERSONNEL**
	• **PLANNED BEHAVIOR**	• **CLINICIANS**
		
• Intend* or intention*	• Behavio?r*	Clinician*
• Inclin* or disinclin*	• Clinician performance*	Counse?lor*
	• (Actor or abstainer) near behavi*r*	Dentist*
		Doctor*
		Family practition*
		General practition*
		GP*/FP*
		Gyn?ecologist*
		H?ematologist*
		Health professional*
		Internist*
		Neurologist*
		Nurse*
		Obstetrician*
		Occupational therapist*
		Optometrist*
		OT*
		P?ediatrician*
		Paramedic*
		Pharmacist*
		Physician*
		Physiotherapist*
		*Primary care*
		Psychiatrist*
		Psychologist*
		Radiologist*
		Social worker*
		Surgeon*/surgery
		Therapist*

Example thesaurus headings are given for the PsycINFO database and were adjusted and exploded as appropriate for other databases.

### Review methods

All titles and abstracts retrieved by electronic searching were downloaded to a Reference Management database; duplicates were removed and the remaining references were screened independently by two reviewers, and those studies which did not meet the inclusion criteria were excluded. Where it was not possible to exclude articles based on title and abstract, full text versions were obtained and their eligibility was assessed by two reviewers. Full text versions of all potentially relevant articles identified from the reference lists of included articles were obtained. The eligibility of each full text article was assessed independently by two reviewers. Disagreements were resolved by discussion or were adjudicated by a third reviewer.

Internal validity was independently assessed by two reviewers on the basis of the level of correspondence between the wording of the intention item and the behaviour as measured (graded as good, poor or unclear), and internal consistency of multiple intention items as measured by Cronbach's alpha [15]. External validity was assessed on the basis of whether the target population was local, regional or national; whether the target population was sampled or whether the entire population was approached; and if the population was sampled, whether it was a valid random (or systematic) sample. Susceptibility to bias was assessed on the basis of the percentage of participants approached for whom the relationship between intention and behaviour was analysed.

For each study, we abstracted, where possible, the Pearson correlation coefficient (r) between the measure of intention and the measure of behaviour (equivalent to the standardised beta coefficient) and its standard error. If this was not available and structural equation modelling had been performed, we abstracted the structural coefficient corresponding to the direct relationship between intention and behaviour and its standard error. If neither a Pearson correlation coefficient nor a structural coefficient was available and multiple regression had been performed, we abstracted the partial correlation coefficient relating the intention to behaviour and its standard error, as well as the model R^2 ^summarizing the proportion of the variance between participants that could be explained by the variables in the regression.

## Results

### Description of the studies

**Figure 1:**
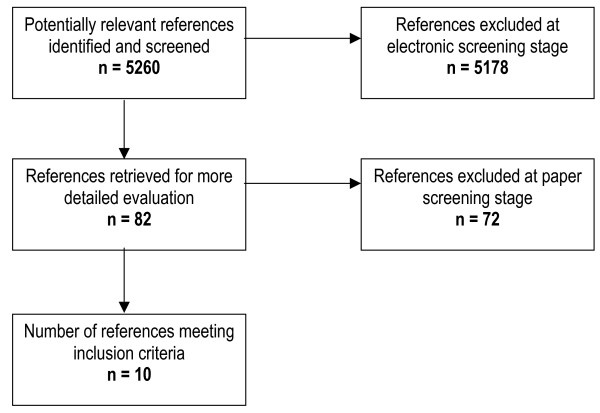
Identification of included references.

The initial searches identified 5260 studies (Fig 1). Of 82 papers retrieved for full text review, 10 fulfilled the eligibility criteria and their data were extracted [16-25]. Reviewers agreed on the eligibility of all the included studies. Of the 72 studies excluded at full text review, 8 (11%) were excluded following reconciliation. [A list of studies excluded at this point is available from the authors.] The eligible studies approached a total of 3777 health care professionals, and data from 1623 (43%) of these were analysed. The characteristics of these studies are presented in Table 2, and further detail is presented in Additional file 1.

**Table 2:** Summary of included study characteristics and results

**Study**	**1. Type of participants** **2. Target population** **3. Sampling strategy**	**Participants approached and analysed**			**1. Theoretical framework** **2. Target behaviour**	**Measure of intention**		**Measure of behaviour**			**Int-Bev corr.**		**Outcome**		
											
		**N**	**n**	**%**		**Description**	**Psy**	**Description**	**Psy**	**Meth**			**beta (SE)**	**p**	**R**^**2**^
			
Millstein^16^	1. Primary care physicians 2. California, USA 3. Stratified random sample from AMA Masterfile	2087	765	(37%)	1. TRA, TPB 2. Patient education	% patients they intended to educate	NA	% patients they educated	NA	SR	Good	TRA: TRB:	0.56^a^ 0.49^a^	< 0.0001 < 0.0001	0.37^b^ 0.40^b^
Farris^17^	1. Community pharmacists 2. All practising in Alberta, Canada 3. Random sample	320	182	(57%)	1. "Theory of goal-oriented behaviour"; included perceived behavioural control 2. Provision of pharmatceutical care activities	2 items, 7 point scale	*	20 items, No. of care activities provided	NA	SR	Good		0.52^c ^(0.11)	< 0.001	-
Godin^18^	1. Nurses 2. One regional hospital, Canada 3. All approached	238	105	(44%)	1. TPB; TIB 2. Adherence to universal precautions for venepuncture	4 items, 7-point scale	0.82^d^	No. of times adhered to universal precautions for last 10 venepunctures performed	NA	SR	Good		0.37	0.001	0.25
Hoppe^19^	1. Primary care nurses 2. 4 districts, UK 3. Random sample of GP practices, one nurse recruited from each practice	260	132	(51%)	1. TRA, TPB 2. Patient education	5 items, 7 point scale	0.91^d^	1 item, 7 point scale	NA	SR	Good		0.56	< 0.001	0.31
O'Boyle^20^	1. Nurses 2. 4 hospitals, USA 3. All approached	474	120	(25%)	1. TPB 2. Adherence to hand hygiene regulations	5 items, 7-point scale	0.74^d^	% times practised hand hygiene	0.94 to 0.98^f^	SR Ob	Unclear Unclear		0.39 0.09	< 0.01 > 0.05	0.15 0.01
Lambert^21^	1. Primary care physicians 2. 5 clinics in one HMO, USA 3. All approached	39	19	(49%)	1. TRA 2. Antibiotic preference	7-point scale for each of 7 drugs	N/A	No. of prescriptions for each drug as % of prescriptions for all 7 drugs	NA	Ob	Unclear		-0.42 to 0.33	All n.s.	0.0 to 0.18
Bernaix^22^	1. Hospital nurses 2. 2 hospitals, USA 3. Sampled – sampling strategy not reported	52	49	(94%)	1. TRA 2. Provision of maternal support	3 items, 7 point scale	0.93^d^	46 items, 5 point scale	0.91 to 0.95^g^	PR	Unclear		*	n.s.	*
Renfroe^23^	1. Hospital nurses 2. 3 hospitals, USA 3. All approached	138	108	(78%)	1. TRA 2. Documentation	2 items, 7 point scale, % patients likely to document	0.66^e^	20 item checklist, No. of items documented	0.71^g ^0.84^h^	D	Poor		0.41 (0.14)	0.003	0.15
Harrell^24^	1. Primary care physicians 2. 11 metropolitan areas, eastern USA 3. Sampled from existing physician panel – sampling strategy not reported	104	93	(89%)	1. TRA 2. Drug preference	7-point scale for each of 5 drugs	N/A	Most frequently prescribed drug	NA	D	Poor		0.27 to 0.52	0.015 to 0.001	0.07 to 0.27
Quinn^25^	1. Nurses 2. General medical and surgical wards of one hospital, USA 3. All working on a specific day	65	50	(77%)	1. TRA 2. Documentation of teaching	1 item, 7 point scale	N/A	No. of patients with documentation of teaching/No. of patients assigned	0.76^f^	D	Good	R1: R2:	0.08 0.02	> 0.05 > 0.05	0.01 0.00

N = Number of participants approached; n = Number of participants analysed; % = Percentage of participants approached who were analysed; Psy = Psychometrics;

Meth = Method of ascertainment of behaviour; Int-Bev Corr = Correspondance between measures of intention and behaviour

* = Not reported; N/A = Not applicable; n.s. = non-significant; SR = Self report; Ob = Observed; PR = Patient report; D = Documented

a Adjusted beta coefficient from multiple regression

b R^2 ^for multiple regression model

c Path coefficient from structural equation modelling

d Cronbach's alpha

e Correlation coefficient

f Inter-rater reliability

g Internal consistency

h Intra-class correlation coefficient

#### Study design

All studies used questionnaire survey methods to elicit intention, and all study designs were non-experimental.

#### Participating healthcare professionals

The number of participants approached in each study ranged from 39 to 2,087 (median 188). In six studies [18-20,22,23,25] the participants were nurses, in three studies they were doctors [16,21,24], and in the one remaining study they were pharmacists [17].

#### Clinical behaviours

The studies covered five types of clinical behaviour: hand hygiene behaviours (e.g., hand washing) [18,20], patient education [16,19,22], clinical record-keeping [23,25], drug choice (prescription) [21,24], and provision of pharmaceutical care [17].

#### Theoretical framework used

The studies reported using mainly the Theory of Reasoned Action (TRA, [26]) [16,19,21-25] and the Theory of Planned Behaviour (TPB, [6]) [16,18-20]. Other frameworks used were Triandis' Theory of Interpersonal Behaviour [27,18] and the "theory of goal-orientated behaviours" [28], with the inclusion of perceived behaviour control [17].

#### Measurement of intention

Six studies used multiple-item intention measures [17-20,22,23], and four studies used single-item intention measures [16,21,24,25], two of which asked the same item for each of a range of drugs [21,24]. The response format for nine studies was a 7-point scale, scored 1–7 [17,20,22,23,25] or scored -3 to +3 [18,19,21,24]. The response format for one study was an estimated percentage, expressed in deciles [16].

#### Measurement of behaviour

One study used patient-report of behaviour [22], four studies used self-report [16-19] one used observation [21], one study used both self-report and observation[20], and three studies used documented data [23-25].

#### Measurement of intention-behaviour relationship

We were able to abstract the correlation between the measures of intention and behaviour for all studies except Bernaix [22]. Only three studies [17,23,24] reported the standard error of this correlation or statistics from which it could be estimated, so it was not possible to aggregate the study results in a meta-analysis.

#### Quality

The reviewers agreed on the correspondence between the intention and behaviour measures for five of the studies, with correspondence ratings for the remaining five agreed upon following reconciliation. Overall 5/10 studies were judged to have good correspondence between the intention and behaviour measures [16-19,25]. Correlations between intention and behaviour ranged from 0.0 to 0.40 when correspondence was "Good," from 0.0 to 0.18 when correspondence was "Unclear," and from 0.07 to 0.27 when correspondence was "Poor."

In 5 of 6 studies where multiple intention items were combined to produce a composite measure of intention internal consistency was acceptable; Cronbach's alphas for measures of intention ranged from 0.74 to 0.93 in four studies [18-20,22], and a correlation co-efficient of 0.66 was reported in one of two studies using two intentions items [23]. No psychometrics were presented for the intention measure used in the remaining study [17].

The psychometric properties presented for four studies using observed behaviour [20], patient reported behaviour [22] and recorded behaviour [23,25] were acceptable; internal consistency ranged from 0.71 to 0.95 for multiple item instruments combined to produce summary scores [22,23], and inter-rater reliability ranged from 0.76 to 0.98 for multiple [20,25] and single-raters [23]. The proportion of participants for whom the relationship between intention and behaviour was analysed ranged from 25% to 94% (median 54%). The one study that used patient-reported behaviour based this on observations by 87% of the observers approached [22].

In terms of external validity, four studies considered regional populations [16,17,19,24]; all other studies considered local populations such as physicians in 5 clinics in one HMO [21], and nurses in one [18], two [22], three [23] or four [20] hospitals. Five studies approached all participants in their target populations [18,20,21,23,25]. Participants in the remaining five studies were sampled – three studies used random selection [16,17,19] and two did not specify their sampling strategy [22,24].

### Relationship between intention and behaviour

The study results are summarised in Table 2 and Additional file 2.

In four of the five studies where a self-reported measure of behaviour was used, the measure of intention corresponded well to the measures of behaviour. All self-report studies, including the one where correspondence was unclear, found a statistically significant correlation between intention and behaviour and R^2 ^ranged from 0.15 to 0.4 [16-20].

In four of the five studies where behaviour was observed, recorded or traceable, the correspondence of the intention and behaviour measures was rated as poor or unclear. Two studies reported correlation coefficients describing the relationship between intention and behaviour for prescribing each of several drugs [21,24]. The estimated correlation between intention and observed, recorded or traceable behaviour ranged from -0.42 to 0.52 (median 0.14) [20,21,23-25]; one study of documentation [23] and one study of drug preference [24] found this relationship to be statistically significant.

Some of the studies may have been too small to find a statistically significant relationship between intention and behaviour, even if such a relationship was present in the population studied. The three smallest studies, [21,22,25] each with an analysed sample of 50 participants or fewer, failed to show a statistically significant relationship.

## Discussion

This review found 10 studies examining the relationship between intention and clinical behaviours in 1623 health professionals. The proportion of variance in behaviour explained by intention was of a similar magnitude to that found in the literature relating to non-health professionals and corresponds to a medium to large effect [29]. This was more consistently the case for studies in which intention-behaviour correspondence was good and behaviour was self-reported for the studies. However, it did also apply to studies with poor correspondence between intention and the measure of behaviour.

The literature assessing the correlation of intention and behaviour in health professionals is small compared to that available for non-health professionals. Sheeran [9], for example, presents data for 82,107 subjects from studies of non-health professionals. The considerably smaller literature for behaviour in health professionals makes it hard to draw firm conclusions based solely on these studies. However, despite the potential constraints on health professional behaviours, this review, viewed in the context of the larger populations of studies, provides encouragement for the contention that there is a predictable relationship between the intentions of a health professional and their subsequent behaviour.

Our review highlights a number of methodological issues within the available literature: the lack of experiments, the methods of measuring behaviour, and the reporting of studies. All of the studies that we found were observational. Unlike Webb [10] we found no reports of experiments that examined the relationship between changes in intention and changes in behaviour. Whilst it is not possible to comment on this relationship in health professionals, it would seem prudent to assume that the same pattern of results would apply and that although we have grounds to support there being medium to large effects in correlational studies, these may well be smaller when the process of change is evaluated within experimental designs.

Our results highlight the major challenge in the practicalities of measuring healthcare professional behaviour. Using self-reported measures of behaviour (compared to measuring actual behaviour) is relatively quick, cheap and easy. Moreover, it allows us to avoid having to deal with the logistics of gathering observed or recorded behaviour data and makes it straightforward to ensure good correspondence between measures of intention and behaviour (a central facet of operationalising theories).

However, healthcare systems are not interested in changing health professionals self-reported behaviour; they want to change actual behaviour in the expectation that this should lead to improved patient outcomes. A major advantage of using theory to design interventions to change the behaviour of health professionals is that it offers a generalisable framework with which to work [30,31]. When building theory-based interventions, outcomes, like intention or self-reported behaviours, can be useful proxies for actual behaviour [5,32]. However, self-reported behaviour has the drawback of not accounting for the range of external factors (e.g. organisational or patients factors) that could be important effect modifiers of a heath professional's behaviour, and any estimate of the potential effect of an intervention based on self-reported behaviour is likely to overestimate its impact [8].

Whilst it may be relatively straight forward to define the behaviours of interest and be able to word a measure of intention, the choice of measures of behaviour can be more problematic. If direct observation is close to a (usually expensive) gold standard then all other methods of measuring behaviour usually involve some degree of compromise, either in terms of the correspondence between the measured behaviour and the behaviour of interest – or in terms of the feasibility of data collection. Three studies in this review that did not find a significant correlation between intention and behaviour did not attempt to measure behaviour through direct observation, but used an alternative measure – number of prescriptions [21], chart review [25], and patient report [22]. These have advantages; for example, two of them are routinely available and therefore relatively cheap to obtain. However, it is more difficult to guarantee the one-to-one correspondence necessary between a measure of intention and a measure of behaviour when the latter is drawn from a potentially unreliable source. Under such circumstances it is not clear whether the absence of a correlation between intention and behaviour is a true lack of correlation or is due to measurement error. For instance, the study using chart review as a data source for teaching behaviour [25] makes the implicit assumption that all teaching activity is recorded, but this may not have been the case.

There was considerable variation and discrepancy in how the studies reported their findings. From the perspective of the systematic reviewer, if no other, it would be helpful if there was an agreed format of presenting the results of such studies. This could relate to issues around the theories used (i.e., clear statements of how each construct was operationalised), study conduct (i.e., clear statement of the timelines relating to administration of measures of intention and behaviour), and analysis (i.e., reporting of Pearson correlations between all variables that are entered into multiple regression analyses).

The findings from this review of health care professionals are broadly consistent with those found in the non-health professional literature. Intention appears to be a valid proxy measure for behaviour for use in the development of implementation interventions. However, there remain significant methodological challenges.

## Competing interests

The author(s) declare that they have no competing interests.

## Authors' contributions

All authors contributed to the conception, design and analysis of the study and approved the submitted draft. ME, JF, EK and SH reviewed the articles and abstracted the data.

## Supplementary Material

Additional file 1 Table 3 Characteristics of included studies. Detailed description of the measures used by the studies included in the review Click here for file

Additional file 2 Table 4: Results. Detailed description of the results reported by each of the studies included in the review Click here for file
