# Supplementary material for: Do self- reported intentions predict clinicians' behaviour: a systematic review
Source: Implement Sci. 2006 Nov 21;1:28. doi: 10.1186/1748-5908-1-28 (PMC1664582; doi:10.1186/1748-5908-1-28)
Supplement: Additional file 1 — Table 3 Characteristics of included studies. Detailed description of the measures used by the studies included in the review [file 1748-5908-1-28-S1.doc]

### Table 3: Characteristics of Included Studies

| **Study** | **Theoretical framework and study aim(s)** | **Setting and participants** | **Intention measure** | **Behaviour measure** |
| --- | --- | --- | --- | --- |
| Millstein [16] | TPB and TRA  **Aims/Research question**  To compare the ability of the TRA and the TPB to predict, prospectively, physicians’ behaviour in regard to educating adolescent patients about the transmission of HIV and other sexually transmitted diseases (STDs). | All primary care physicians who were board certified in family practice, paediatrics, obstetrics and gynaecology or internal medicine in California, USA.  **Inclusion/ exclusion criteria**  Actively practising physicians who spent at least 50% of their time in direct patient care, and saw sufficient numbers of adolescent patients in routine visits. | One item: Physicians were asked to assess the percentage of adolescent patients they intended to educate over the next five to six months.  **Response format**  Percentage estimates – scale presented in deciles. | Participants were asked to report on their behaviour over the preceding six months using the same response scale as for intention.  **Response format**  Percentage estimates – scale presented in deciles.  **When measured**  Six months after measure of intention. |
| Farris [17] | “Theory of goal-orientated behaviours” and included PBC  **Aims/Research question**  1. To describe self-efficacy, beliefs, evaluation and PBC in the provision of pharmaceutical care.  2. To quantify intention and behaviour to promote pharmaceutical care in a 2-week period.  3. To examine the relationship between intention and behaviour. | Community pharmacists from Alberta Pharmaceutical Association. Canada.  **Inclusion/exclusion criteria:**  None. | Two items: Pharmacists were asked to rate their intention to provide all (20) pharmaceutical care activities for one patient during next two weeks.  1. I intend to try to provide all pharmaceutical care activities for one patient in my practice in the next two weeks.  2. During the next two weeks, I plan to try to provide all pharmaceutical care activities for one patient in my practice.  **Response format:**  Extremely unlikely / disagree to extremely likely / agree;  7-point scale, scored 1 to 7.  **Summary statistic**  Mean score. | Pharmacists were asked to indicate on a self-report questionnaire (20 Items) whether or not they had completed the 20 behaviours for the one patient they had provided the most pharmaceutical care for during the past two weeks.  **Response format**  Yes / No  **When measured**  Two weeks after intention. |
| Godin [18] | TPB, Triandis’ Theory of Interpersonal Behaviour.  **Aims/Research question**  To predict and explain nurses’ adherence to universal precautions (UPs): hand washing, use of gloves, correct handling/disposal of needles) when performing venipuncture (VP). | All Hospital nurses in one secondary care regional hospital in Canada.  **Inclusion/exclusion criteria**  Excluded if no contact with patients. | Four items: During the next 3 months:  I have the intention of adhering to UPs; I will adhere to UPs; If I can, I will adhere to UPs; My chances that I will adhere to UPs.  **Response format**  Unlikely to, likely, or low to high;  7-point scale, scored -3 to +3.  **Summary statistic**  Mean score.  **Psychometrics**  Cronbach’s alpha = 0.82 | Nurses were asked to estimate how many times they had adhered to UPs for the last 10 VPs performed in past three months.  **Response format:**  n/10 VPs for which they had adhered to UPs in last three months.  **When measured**  Three months after measurement of intention. |
| Hoppe [19] | TRA and TPB  **Aims/Research question**  To examine practice nurses’ decisions and reported behaviour regarding raising the issue of weight loss with overweight patients using the TRA/TPB. | Primary care practice nurses from four administrative districts (FHSAs) in the UK.  **Inclusion/exclusion criteria:**  None reported. | Five items: I intend to raise the issue of  weight loss; I plan to raise the issue of  weight loss; I want to raise the issue of  weight loss; I am committed to raising  the issue of weight loss; How likely is it  that you will raise the issue of weight?  **Response format**  1 & 2: Definitely do to Definitely do not  3 & 4: Strongly agree to Strongly disagree  5: Extremely likely to Extremely unlikely.  7-point scale, scored -3 to +3.  **Summary statistic**  Mean score on 5 items.  **Psychometrics**  Cronbach’s alpha = 0.91. | During the last month, I have raised the issue of weight loss with all overweight patients.  **Response format**  Very infrequently to Very frequently;  7 point scale, scored -3 to +3.  **When measured**  Four weeks after measurement of intention. |
| O’Boyle [20] | TPB  **Aims/Research question**  1.) Estimate overall adherence to hand hygiene recommendations.  2.) Describe relationships among variables from TPB, self-reported adherence, observed adherence, and level of activity in the nursing unit. | All Nurses working in medical/surgical intensive care units and the associated post-intensive care units of four community, non-profit teaching hospitals in mid-west, USA from July 1996 to October 1997.  **Inclusion/ exclusion criteria**  Included if they were Staff or Charge Nurses who provide direct patient care, were employed for at least 6 months, and worked an average of at least 1 day per week. | Five items: Nurse’s plan to adhere to hand hygiene recommendations in a variety of clinical situations  **Response format**  Extremely unlikely to extremely likely;  7-point scale, scored 1 to 7.  **Summary statistic**  Mean score.  **Psychometrics**  Cronbach’s alpha = 0.74 | ***(Self report)*** Nurses asked to estimate the percentage of times (from zero to 100%) that they had practised hand hygiene.  **Response format**  Percentage estimate.  ***(Observed)*** Observers recorded the number of indications for hand-washing and the number of times nurses washed their hands, when the indications arose. Nurses were observed until 120 minutes or 10 hand-washing indications were obtained.  **Response format**  Hand-washing score: Hand-washing events/Hand-washing indications x 100. **Psychometrics**  **Inter-rater reliabilities (from pilot study)** 0.94 & 0.98.  **When measured**  2 weeks to 4 months after measurement of intention. |
| Lambert [21] | TRA  **Aims/Research question**  Hypothesis: Antibiotic prescribing behaviour will be significantly correlated  with stated intention to prescribe. | Family Practice or internal medicine physicians in 5 Primary Care clinics in one HMO, USA.  **Inclusion/exclusion criteria**  None reported. | One item: Participants were asked to rate the statement, “When <drug>is one of several indicated alternatives, I intend to prescribe <drug>” for each of seven antibiotics.  **Response format**  “Extremely unlikely” to “extremely likely”, 7-point scale, scored -3 to +3. | Number of prescriptions issued during 4^th^ quarter 1994.  **When measured**  4^th^ quarter 1994, less than 6 months.  **Summary statistic**  Proportion of total number of prescriptions written by each physician for all 7 antibiotics combined. |
| Bernaix [22] | TRA  **Aims/Research question**  To identify the external factors and characteristics of nurses that influence their ability to provide effective informational, technical and emotional support to breast-feeding mother’s and their infants.  What are the relationships between postpartum and nursery nurses’:  1. Attitudes, subjective norms, demographics, knowledge and behavioural intentions.  2. Intentions to provide support to mothers and actual provision of that support. | Nurses and postpartum, breast-feeding, mothers. Two metropolitan teaching hospitals in Mid-Western region of USA.  **Inclusion/exclusion criteria**  Included registered nurses with minimum 6 months experience in any post-partum or newborn setting.  Post-partum mothers, breast-feeding with minimum 8 hours care from Phase 1 nurse.  Excluded: mothers of newborns <37 weeks gestation. | Three items: Participants were asked to rate the statements “I would rate my intention to provide (technical, emotional, informational) support to breast-feeding mothers I am assigned as…”  **Response format**  Very weak to very strong;  7-point scale, scored 1 to 7.  **Summary statistic**  Mean score.  **Psychometrics**  Cronbach’s alpha = 0.93. | Patient report: Informational support (16 items), Technical support (15 items) and Emotional support (15 items)  **Response format**  Strongly agree/strongly disagree;  5-point scale.  **When measured**  2 weeks after baseline.  **Summary statistic**  For each patient: Summary “total support score” across 46 items.  For each nurse: The mean score of the patient summary scores.  **Psychometrics**  Cronbach’s alpha=0.91 to 0.95. |
| Renfroe [23] | TRA  **Aims/Research question**  To develop and test an exploratory model that explains the behaviour of documentation by nurses. | All staff nurses on all units within three hospitals in the south east USA.  **Inclusion/exclusion criteria**  None. | Two items: Participants were asked to:   1. Give % estimate of 2. Rate the likelihood that they would document optimally on all patients.   **Response format**  1. % estimate.  2. 7-point scale, scored (not stated)  **Psychometrics**  Correlation co-efficient = 0.66 | 20-item instrument covering: nursing assessment, interventions, evaluation and mechanics of documentation, (e.g. noting date, time, correct spelling, and approved abbreviations).  One patient’s chart was randomly selected for each nurse’s shift. If admitted on that shift or had discharge orders, another patient’s record was selected for evaluation.  **Response format**  Checklist and accompanying guidelines were used, and each item was scored as present or absent.  Based on what should be documented in any hospital patient’s chart on an 8-hour shift.  **When measured**  After the shift before which intention was measured.  **Summary statistic**  Unclear.  **Psychometrics**  Unclear – Kuder-Richardson–20 for internal consistency was 0.71. Intraclass Correlation Coefficient = 0.84 for a single rater. |
| Harrell [24] | Fishbein’s Behavioural intentions model  **Aims/Research question**  Specific research objectives:  1) To provide measurement for brand choice prediction based on opinion interviewing;  2) To clarify the relationships among theoretical constructs “attitudes” “beliefs” “behavioural intentions” and “behaviour”, as they apply to purchase situations. | Private internists and GPs currently practising in primary and secondary care community settings in 11 metropolitan areas in eastern half of USA.  **Inclusion/exclusion criteria**  None. | One item: Participants were asked to rate the statement for each of 5 different drugs: “Please give your drug preference for treating this patient. That is, when a similar prescribing situation occurs, what is the probability that you would choose each drug?”  Physicians were asked to place their decision to prescribe a certain brand of drug in the context of a case simulation designed to represent a typical case of maturity onset diabetes.  **Response format**  Extremely improbable to extremely probable;  7-point scale, scored -3 to +3. | Physician Panel Data: Carbon copy of actual prescribing data for each physician by total prescriptions and brand for each month.  **Response format**  A score of one for the brand used most often by the doctor and zero for all other brands.  **When measured**  Behaviour data was collected after intention measure and consisted of 3 months prescribing behaviour for each physician - but not stated if 3 months from measurement of intention.  **Summary statistic**  Brand used most often by doctors |
| Quinn [25] | TRA  **Aims/Research question**  Hypothesis: There will be no relationship between the documentation behaviour with respect to patient teaching and behavioural intention of registered nurses. | Registered nurses in all general medical and general surgical units in a 250 bed hospital, USA.  **Inclusion/exclusion criteria**  Nurses working on one particular day. | One item: Participants were asked to rate the statement “At the end of the work day, I intend to document the type of patient teaching I carry out.”  **Response format**  “extremely improbable or unimportant” to “extremely probable or important”; 7-point scale, scored 1 to 7 | Documenting on a flow chart instances of addressing patient teaching. Charts were reviewed for documentation on the same day that intention was assessed by questionnaire.  **Response format**  Presence of documentation of any teaching for an individual patient.  **When measured**  Data were collected from December 1994 through February 1995.  **Summary statistic**  Any documentation of teaching (Y/N) divided by the number of allocated patients.  **Psychometrics**  Data collected by the investigator and 5 second-raters. Inter-rater correlation = 0.759 |
