# Supplementary material for: Do self- reported intentions predict clinicians' behaviour: a systematic review
Source: Implement Sci. 2006 Nov 21;1:28. doi: 10.1186/1748-5908-1-28 (PMC1664582; doi:10.1186/1748-5908-1-28)
Supplement: Additional file 2 — Table 4: Results. Detailed description of the results reported by each of the studies included in the review [file 1748-5908-1-28-S2.doc]

**Table 4: Results**

| **Study** | **Sample** | **Method of measuring behaviour** | I: Mean (SD)  B: Mean (SD)  Correspondence of measures of intention and behaviour | **Correlation of Intention/Behaviour**  r beta r*^2^* *p-value* |
| --- | --- | --- | --- | --- |
| Millstein 1996 [16] | Approached: 2087  Analysed: 786  % analysed: 37  **Age:** Not reported  **Gender:** 64% male (of 786) | Self-report | I: Not reported  B:Not reported  Correspondence: Good | 0.61 0.56 0.37 <0.01 **TRA Model**  0.63 0.49 0.40 <0.01 TPB Model |
| Farris 1999 [17] | Approached: 320  Analysed: 182  % analysed: 57  **Age:**Not reported  **Gender:** 41% male | Self-report | I: 5.0 (1.5)  B: 9.5 (3.7)  Correspondence: Good | **0.52** <0.001 |
| Godin 2000 [18] | Approached: 238  **Age:**20-49 yrs (for 92% respondents)  **Gender:**8% male  Analysed: 105  % analysed: 44 | Self-report | I: 2.26 (0.83)  B: 5.60 (3.19)  Correspondence: Good | **0.37 0.25 <0.001** |
| Hoppe 1999 [19] | Approached: 260  Analysed: 132  % analysed: 51  **Age(yrs: mean, (sd), range)**  42.8 (8.8), 27-63  **Gender:** 0% male | Self-report | I: 1.0 (1.25)  B: 0.91 (1.56)  Correspondence: Good | 0.56 **0.56 0.31 <0.001** |
| O’Boyle 2001 [20] | Approached: 474  Analysed: 120  % analysed: 25  **Age/Gender**  Not reported | Self-report  Observed | I: 5.93 (0.76)  B: 81.70 (15.68) (Self-report)  Correspondence: Unclear (self-report)  B: 70.0 (0.22) (Observed)  Correspondence: Unclear (observed) | 0.09 0.09 0.008 >0.05  0.385 0.385 0.25 <0.05 |
| Lambert 1997 [21] | Approached: 39  **Age(yrs: mean, (sd))**  41.6 (7.8)  **Gender:** 44% male  Analysed: 19  % analysed: 49 |  | Intention Behaviour  Mean (SD)  Amoxicillin 2.12 (1.27) 0.09 (0.13)  Co-amxoiclav -0.32 (2.39) 0.07 (0.10)  Clarythromycin 0.32 (2.08) 0.15 (0.14)  Cefaclor -0.52 (2.10) 0.09 (0.25)  **Cefuroxime** -0.32 (2.15) 0.03 (0.07)  Erythromycin 0.04 (2.17) 0.10 (0.11)  Co-trimoxazole 1.88 (1.48) 0.47 (0.26)  Correspondence: Unclear | r p-value  Amoxicillin -0.42 ns  Co-amxoiclav 0.06 ns  Clarythromycin -0.01 ns  Cefaclor 0.07 ns  **Cefuroxime** 0.14 ns  Erythromycin -0.23 ns  Co-trimoxazole 0.33 ns |
| Bernaix 2000 [22] | Approached: 52  Analysed: 49  % analysed: 94  **Age(yrs: mean, (sd), range)**  *Nurses:* 38.9 (9.6), 23-62  *Mothers:* 29.9 (5.1), 16-44  **Gender: 1**00% female | Patient report | I: 17.8 (3.0) (Nurses)  B: 181 (30) (Mothers)  Correspondence: Unclear | Not reported |
| Renfroe 1990 [23] | Approached: 138  Analysed: 108  % analysed: 78  **Age(yrs: mean, (sd))**  33.3 (9.5)  **Gender:** 5% male |  | I: Not reported  B: 7.2 patients documented per shift  Correspondence: Poor | 0.22 0.15 (strength of intention)  0.31 (% estimate of intention)  0.41 0.003 (path coefficient) |
| Harrell 1974 [24] | Approached: 104  Analysed: 93  % analysed: 89  **Age/gender**  Not reported |  | I: Not reported  B: Not reported  Correspondence: Poor | 0.52  **0.52 0.27** not reported Drug A  0.47 **0.47 0.22** not reported Drug B  0.27 0.27 **0.07** not reported Drug C  0.27 **0.27 0.07** not reported Drug D  0.34 0.34 **0.11** not reported Drug E  0.40 - - - Average (A-E) |
| Quinn 1996 [25] | Approached: 65  Analysed: 50  % analysed: 77  **Age(yrs: mean, (sd), range)**  35 (7.8), 21-60 (missing for 3 participants)  **Gender:**Not reported |  | I:1.44 (1.37)  B: Not reported  Correspondence: Good | 0.082 0.082 0.01 >0.05 Rater 1 (Investigator)  0.020 0.020 0.00 >0.05 Rater 2 (Other) |
